# A Reevaluation of the Tolerability and Effects of Single-Dose Ivermectin Treatment on *Onchocerca volvulus* Microfilariae in the Skin and Eyes in Eastern Ghana

**DOI:** 10.4269/ajtmh.21-0859

**Published:** 2021-11-29

**Authors:** Nicholas O. Opoku, Michael E. Gyasi, Felix Doe, Daphne Lew, Augustine R. Hong, Sithembele Chithenga, Peter U. Fischer, Christopher L. King, Gary J. Weil

**Affiliations:** ^1^ School of Public Health, University of Health and Allied Sciences, Ho, Ghana;; ^2^ St. Thomas Eye Hospital, Accra, Ghana;; ^3^ Hohoe Municipal Hospital, Hohoe, Ghana;; ^4^ Division of Biostatistics, Washington University School of Medicine, St. Louis, Missouri;; ^5^ Department of Ophthalmology, Washington University School of Medicine, St. Louis, Missouri;; ^6^ Infectious Diseases Division, Department of Medicine, Washington University School of Medicine, St. Louis, Missouri;; ^7^ Center for Global Health and Diseases, Case-Western Reserve University, Cleveland, Ohio

## Abstract

Mass administration of ivermectin (IVM) has significantly reduced onchocerciasis prevalence, intensity, and morbidity in most endemic areas. Most IVM clinical trials were performed long ago in persons with high-intensity infections that are uncommon in West Africa today. This cohort treatment study recruited participants from a hypoendemic area in eastern Ghana to reevaluate the efficacy and tolerability of IVM with a special focus on the kinetics of microfilaria (Mf) clearance. Mf in the skin and anterior chambers (AC) were assessed by skin snip and slit lamp examinations at baseline and at 3 and 6 months after treatment with IVM 150 μg/kg. Most participants (184–231, 79.7%) enrolled were treatment-naïve. The baseline geometric mean skin Mf count was 12.67/mg (range 3–86). Although persons with MfAC at baseline (64/231, 27%) had significantly higher skin Mf counts than people without MfAC, 7 of 39 (15%) of persons with skin Mf counts in the range of 3–5 Mf/mg had MfAC. Skin Mf were detected in 14% (31/218) and 45% (96/216) of participants 3 and 6 months after IVM treatment, respectively. MfAC were detected in 12 of 212 (5.7%) study participants at 6 months. 81% (187 of 231) of participants experienced 439 adverse events within 7 days after treatment; all adverse events were mild (96.1%) or moderate. This study has provided new data on the kinetics of Mf in the skin and eyes after IVM treatment of persons with light to moderate intensity *Onchocerca volvulus* infections that are common in Africa at this time.

## INTRODUCTION

Onchocerciasis is a parasitic infection that is caused by the filarial nematode *Onchocerca volvulus* and transmitted by the bite of *Simulium* blackflies that breed in rivers. Larval parasites (microfilariae or Mf) migrate through the skin and can cause severe cutaneous and ocular disease (“river blindness”). The World Health Organization (WHO) estimates that at least 25 million people are infected, most of whom reside in 31 sub-Saharan African countries.^1^^,^^2^ Onchocerciasis is the world’s second leading infectious cause of blindness. An estimated one million people have severe visual impairment or blindness because of onchocerciasis. Mass drug administration (MDA) with ivermectin (IVM) for onchocerciasis has significantly reduced the burden of infection to the point where severe ocular disease is now uncommon in most endemic areas.^2^ However, because IVM has no permanent effect on adult worms that can live for more than 10 years, the use of IVM alone for onchocerciasis elimination has been most successful in areas with low transmission or in focal transmission zones in the Americas^3^ Senegal and Mali^4^ or Northern Sudan.^5^ Other groups have reported that 10 or more rounds of IVM were not sufficient to interrupt transmission in some areas.^6^^–^^8^ Thus, there is an urgent need to find new drugs or novel combinations of existing drugs that can kill or sterilize adult worms if onchocerciasis is to be eliminated within a reasonable time frame.^9^^,^^10^

Most studies of IVM in onchocerciasis were performed long ago in persons with high intensity infections that are rarely seen today.^11^^,^^12^ Recent studies have suggested that IVM may have suboptimal activity against *O. volvulus* in savannah regions in Ghana.^13^ Therefore, one goal of this study was to reevaluate the efficacy and tolerability of IVM for treatment of onchocerciasis in Ghana with a special focus on the kinetics of Mf clearance from the skin and eyes. Ophthalmological monitoring included optical coherence tomography (OCT),^14^ which had not previously been used in patients with onchocerciasis. Another goal of this study was to identify participants for a planned future study of a new treatment of onchocerciasis that requires pretreatment with IVM to reduce Mf counts.

## METHODS

### Ethical approval, registration, and study protocol.

This study is registered at CLINICALTRIALS.gov (NCT03517462). The full study protocol is provided as S1 Appendix to this paper. Briefly the primary objective of the study was to determine the proportion of participants who had complete Mf clearance from the eye at 3 and 6 months following IVM treatment. Secondary objectives were to assess the kinetics of the clearance of Mf from the eyes and skin after IVM treatment and to assess the utility of ocular coherence tomography (OCT) for evaluating ocular tissues and intraocular parasites in persons with onchocerciasis. The protocol was reviewed and approved by ethical review committees at the University of Health and Allied Sciences (UHAS) in Ho, Ghana, the Ghana Health Service, Case-Western Reserve University (Cleveland, OH) and Washington University School of Medicine (St. Louis, MO).

### Inclusion and exclusion criteria.

Inclusion criteria included persons between the ages of 16 and 70 with at least one palpable subcutaneous nodule (onchocercoma) and ≥ 1 Mf/mg of skin. Persons who met those criteria were excluded if they were pregnant or breastfeeding mothers within one month of delivery, had a history of allergy or intolerance to IVM, or had been treated with IVM within the past six months. The protocol also excluded persons with serious baseline ocular disease such as glaucoma, severe keratitis, uveitis, or cataracts that interfered with visualization of the posterior segment of the eye.

### Screening and participant enrollment.

Screening and recruitment were performed in Nkwanta North District in the Volta region of Ghana. Participants were from villages that are hypoendemic for onchocerciasis (nodule prevalence < 20%), and mass administration of IVM had not yet been implemented in these villages. The study team met with community leaders and local health personnel in the study area and held open community meetings to explain the purposes and plans for the study prior to screening and recruitment of participants. The meetings and consent forms were in English and a local language (either Twi or Ewe) understood in the study area. Participation required written consent for adults and written consent from a parent or guardian plus assent for minors less than 18 years of age.

Persons were initially screened for the presence of onchocercal nodules by manual palpation. Persons with nodules were tested for microfiladermia as follows. Four skin snips were collected (one from each posterior iliac crest and each posterior calf) with a Holth corneoscleral punch. Snips were weighed and incubated in 100 μL of isotonic saline in individual wells of a flat-bottomed microtitre plate at ambient temperature for at least 8 hours. Snips were then examined by indirect microscopy, and Mf were counted by experienced microscopists. Mean values for Mf/mg for four snips were calculated.

### Medical history and physical examinations.

A brief medical history reviewed prior illnesses and current medications. A review of systems was performed to identify baseline symptoms with special attention to any history of prior onchocercal eye or skin disease or treatment. The physical examination included height, weight, and vital signs with special attention to skin lesions and lymph nodes.

### Ophthalmological examinations.

Study participants were evaluated with a panel of tests two days prior to IVM treatment. Details are provided in the protocol (Supplemental File 1). Briefly, the panel included tests of visual acuity, visual fields, pupillary reflex, applanation tonometry, indirect ophthalmoscopy, fundus photography and optical coherence tomography (OCT), which provides detailed images of the posterior segment including the retina. Slit lamp examinations were performed to assess ocular abnormalities in the cornea and anterior segment. Participants sat with their heads bent as far forward and down for at least three minutes prior to the slit lamp examination to optimize visualization of Mf in the anterior chamber (AC).

### Drug treatment, adverse event (AE) assessments, and follow-up.

Eligible, consenting study participants were treated with 150 μg/kg of IVM *per os* under direct observation. Participants were housed in the UHAS School of Public Health Research Center, which is located within the grounds of the Hohoe Government Hospital in Hohoe, Ghana. Participants were evaluated daily for 7 days after treatment and asked whether they had symptoms suggestive of systemic (e.g., fever, headache), cutaneous, or ocular AEs. A study physician performed a directed physical examination for all participants. All participants had a full ophthalmological examination as described above on day 7 after IVM treatment. Skin snip tests and the full battery of ophthalmological tests were repeated at 3 and 6 months after IVM treatment.

### Data management.

Participant data were recorded on paper case report forms and later entered into REDCap at the UHAS School of Public Health; that data center also validated and cleaned the data. REDCap files included participant’s study identification numbers without personal identifiers. A parallel participant key linked study ID numbers with personal identifying information (name, date of birth). Encrypted REDCap data were transferred to a dedicated server housed at Washington University in St. Louis. A data manager at Washington University performed additional data cleaning and validation and communicated with the UHAS data manager and study investigators to clear queries prior to data lock.

### Statistical methods.

Descriptive statistics were calculated as frequencies and proportions for categorical variables, medians and interquartile ranges (IQRs) for continuous variables, and geometric means and ranges for highly skewed continuous variables. All correlations between variables of interest were calculated using Spearman rank correlations. Additional statistical comparisons were performed using χ^2^ and Fisher’s exact tests for categorical variables, *t*-tests for continuous variables, and Mann–Whitney *U* tests for skewed continuous variables. The total ocular Mf count (MfAC) was calculated by summing the numbers of Mf identified in the AC of each eye. All data analysis was conducted in SAS for Windows version 9.4 (SAS Institute, Cary, NC), and α = 0.05 was used to determine statistical significance.

## RESULTS

### Enrollment and treatment.

A total of 1,030 subjects who were screened near Kpassa (the capital of Nkwanta North district) had at least one palpable subcutaneous nodule. Of these, 274 with at least 1 Mf/mg of skin were invited to Hohoe (170 km south of Kpassa) for secondary screening. Thirteen people were not able to make the trip. Thirty people were excluded because of severe ocular disease, other significant comorbidities or pregnancy. Characteristics of the study sample with skin Mf data over time are presented in Table 1. Thus, 231 people were enrolled into the study with full baseline examinations and treatment with IVM. Most participants (184/231, 79.7%) were treatment-naïve prior to this study (no prior IVM). Only 27 of 231 (11.7%) of study participants had received IVM in the 3 years prior to our study.

**Table 1:** Descriptive characteristics of study participants and skin snip Mf data over time*

Variable	Group	Descriptive statistics
Gender	Female	92 (39.8%)
	Male	139 (60.2%)
Age at baseline (years)		39.1 (29.2, 52.4) [231]
BMI at baseline (kg/m^2^)		19.6 (18.5, 21.3) [229]
Mean skin Snip Mf count (Mf/mg)	Baseline	12.66 (3, 86.29) [231]
	3 months	0.07 (0, 11.28) [218]
	6 months	0.31 (0, 5.74) [212]
Mf prevalence (any snip)	Baseline	231/231 (100%)
	3 months	31/218 (14.2%)
	6 months	96/212 (45.3%)

*Gender and skin snip positivity for microfilariae (Mf) are reported as n (%). Numbers of participants are shown in brackets. Age and BMI are reported as median (IQR), and skin snip Mf counts are reported as geometric mean (range).

### Mf counts in the skin and eyes before and after treatment.

Baseline and follow-up skin Mf data are summarized in Table 1. The median number of palpated nodules per participant at baseline was 2 (IQR: 1, 3), and skin Mf counts were significantly correlated with nodule number (Spearman rank correlation 0.191, *P* = 0.004). Skin Mf counts in persons who had received IVM in the past 3 years tended to be lower than those in people who had no treatment in the past 3 years. Geometric means and ranges were 9.0 (3–49) versus 13.2 (3–86), respectively (*P* = 0.036). Ocular Mf were less commonly seen in persons who had received IVM in the past 3 years, but the difference was not statistically significant (60/204 [29.4%] versus 2/27, [14.8%], *P* = 0.11).

Most participants were restudied at 3 and 6 months (95.7% and 91.8%, respectively). Skin Mf prevalence and densities were dramatically reduced after IVM treatment. Most study participants had negative skin snips 7 days after treatment (data not shown). Although the geometric mean Mf density in skin snips was reduced by 95.7% from baseline 6 months after IVM treatment, 45.3% of 212 participants tested had Mf in skin snips at that time. Baseline skin snip Mf counts were higher in participants with positive skin snips at 6 months than in those with negative skin snips at that time (geometric means, 17.1 versus 8.85, *P* < 0.001).

**Figure 1.**
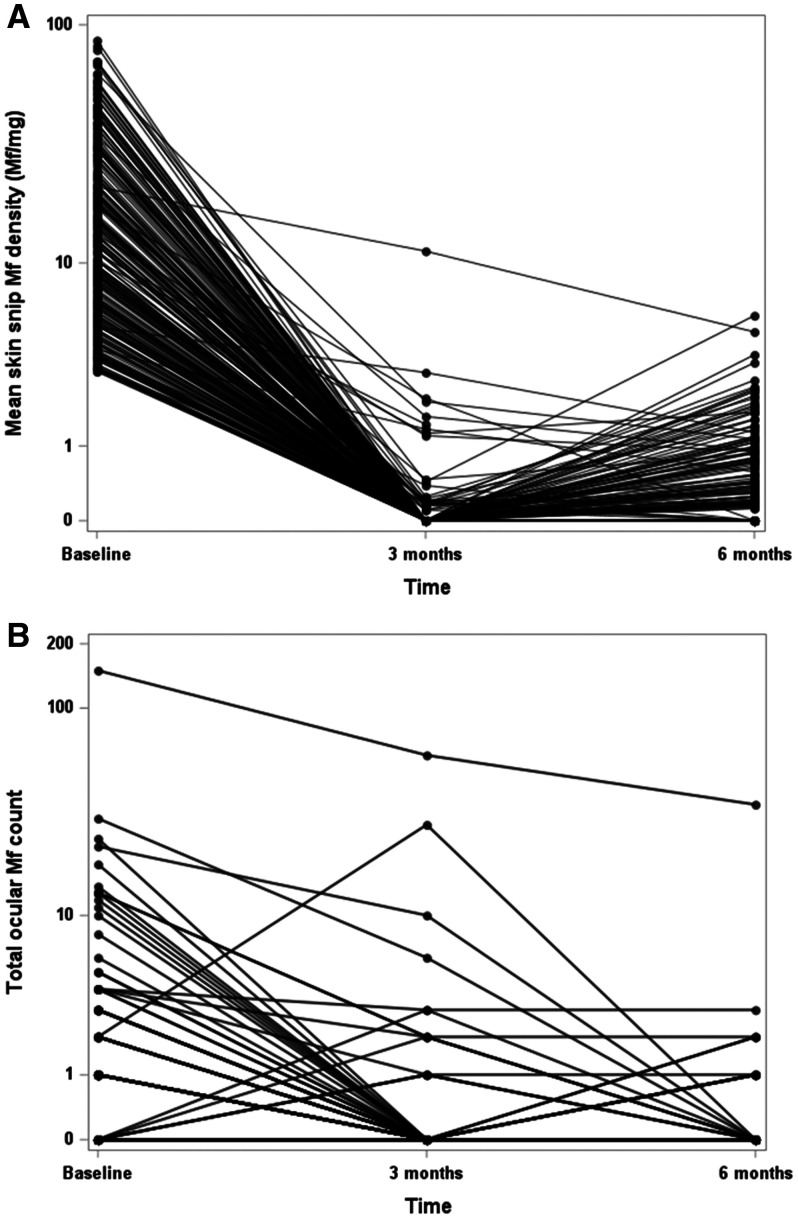
Microfilaria (Mf) counts in skin (**A**) and eyes (**B**) in individuals at baseline and after ivermectin treatment.

Changes in skin snip Mf counts in individual participants after IVM treatment are shown in Figure 1A. Note the Log scale for Mf counts. The figure shows that many people with negative skin snips at 3 months had Mf detected at 6 months. It also shows that skin Mf counts decreased only slightly after IVM treatment in three participants (1.4% of 212 persons tested 6 months after treatment).

## OCULAR FINDINGS BEFORE AND AFTER TREATMENT

No Mf were detected in the posterior segment in any participants’ eyes by any method, and no corneal Mf were seen. Baseline ocular disease in enrolled participants identified without OCT included macular scarring (*N* = 10, 4.3%), cataract (*N* = 3, 1.3%), optic atrophy *N* = 1, 0.43%), and other (2.1%). Sixteen persons (6.9% of 231 participants) had findings that were considered suspicious but non-diagnostic for glaucoma; no participant had elevated intraocular pressure. Baseline OCT findings in the enrolled subjects included chorioretinal atrophy and scarring (*N* = 15), macular drusen (*N* = 8, 3.5%), epiretinal membrane (*N* = 1), cystoid macular edema (*N* = 1). More details on opthalmological abnormalities will be reported separately.

Ocular Mf data before and after treatment are shown in Table 2 and Figure 1B. Sixty-four (27.7%) of study participants had Mf visible in the anterior chamber (MfAC) detected by slit-lamp examination in one or both eyes at baseline. The median time that participants held their heads down prior to examination was 12 minutes (IQR: 10–17). There was a weak correlation between time with head down and baseline MfAC counts (Spearman rank correlation 0.09, *P* = 0.176). The MfAC data include results from one outlier who had very high MfAC counts before and after treatment. No other participant had a MfAC count > 3 (total for both eyes) at 6 months. Percentages of participants with more than 5 Mf visible in either AC were 7.8%, 10.8%, 1.8%, and 0.5% at baseline, 7 days, 3 months, and 6 months after treatment, respectively. Three participants with negative MfAC at baseline had MfAC 6 months after treatment. Two of these persons had MfAC on day 7, but one had no MfAC detected at baseline or day 7.

**Table 2:** Prevalence and densities for ocular microfilariae in the anterior chamber (MfAC) before and after ivermectin treatment

	Left eye		Right eye		One or both eyes (sum)	
Time point	*n*/*N* (%)	Geometric mean (range)	*n*/*N* (%)	Geometric mean (range)	*n*/*N* (%)	Geometric mean (range)
Baseline	45/231 (19.5%)	2.67 (1, 50)	47/231 (20.3%)	2.9 (1, 100)	64/231 (27.7%)	3.4 (1, 150)
7 days	55/231 (23.8%)	2.5 (1, 30)	54/231 (23.4%)	3.58 (1, 80)	73/231 (31.6%)	3.89 (1, 82)
3 months	5/218 (2.3%)	8.37 (3, 20)	12/218 (5.5%)	2.73 (1, 40)	14/218 (6.4%)	3.73 (1, 60)
6 months	8/212 (3.8%)	1.73 (1, 15)	7/212 (3.3%)	2.09 (1, 20)	12/212 (5.7%)	1.98 (1, 35)

Geometric mean and range calculations only considered data from individuals with MfAC detected.

MfAC at baseline were no more common in males than in females (*P* = 0.88). Baseline skin Mf counts (Mf/mg) for persons without and with MfAC at baseline were 10.6 (range 3–68) and 20.0 (3–86.3), respectively (*P* < 0.001). More details on the frequency of MfAC by skin snip Mf count are provided in Table 3. MfAC were observed seven days after treatment in 32 of 167 (19.2%) persons with no ocular Mf detected at baseline. Thus, 96 of 231 (41.6%) of study participants had MfAC detected by slit lamp examinations at baseline and/or on day 7. It was also interesting that 23 of 64 (35.9%) persons with MfAC detected at baseline had no MfAC detected on day 7.

**Table 3:** Baseline frequency of MfAC by mean skin snip Mf/mg

	Frequency (%) of MfAC in either eye		
Mean skin snip Mf/mg	No	Yes	Total
3–5	39	7 (15.2)	46
> 5 to 10	50	11 (18.0)	61
> 10 to 20	41	12 (22.6)	53
> 20 to 30	13	8 (38.1)	21
> 30	24	26 (52)	50
Total	167	64 (27.7)	231

MfAC. Microfilaria(e) (Mf) present in either anterior chamber detected by slit lamp examination.

MfAC prevalences and densities decreased significantly at the 3- and 6-month time points in most study participants after IVM treatment (Table 2 and Figure 1B) (*P* < 0.01 for both comparisons). However, MfAC were detected in 12 of 212 (5.7%) study participants who were examined at 6 months; 3 persons with low MfAC counts at 6 months did not have MfAC recorded at baseline. Two of these persons had MfAC detected 7 days after treatment. The person with 150 MfAC at baseline had 35 MfAC at 6 months; the other 11 persons with MfAC at 6 months had MfAC values between 1 and 3 at that time. A univariable analysis showed that baseline skin Mf counts, presence of MfAC, and MfAC counts (sum for both eyes) at baseline were all significant risk factors for MfAC 6 months after IVM treatment (*P* = 0.01, 0.005, and < 0.001, respectively). However, the presence of MfAC at 6 months was not significantly more common in persons with positive skin snips at that time.

### Adverse events recorded during the first 7 days after IVM treatment.

Early onset AEs are listed in Supplemental Table 1. One or more adverse events were recorded for 187 of 231 participants (81%) within 7 days of treatment. A total of 439 separate AEs were recorded during this 7-day period. Of these, 422 were mild (Grade 1) and 17 were moderate (Grade 2). No severe or serious AEs were recorded. Most AEs were mild or moderate Mazzotti reactions. The most common general (non-ocular) AEs were itching skin (40.3%), headache (23.4%), and joint or muscle pain (19.5%). The most common ocular AEs were itching (6.9%) and pain (4.3%); all ocular AEs were Grade 1. Most ocular and general AEs occurred by day 1 after treatment, and they were of short duration; few AEs were recorded after day 3, and none on day 7. A univariable analysis showed that none of the baseline characteristics considered (sex, age, BMI, skin Mf count, and MfAC) were significant risk factors for AEs within 7 days after IVM treatment.

### Adverse events at later time points.

AEs recorded during participant evaluations performed at 3 and 6 months after treatment are listed in Supplemental Table 2. Almost 58.5% of participants had one or more AEs recorded at these time points. The most common AE was ocular itching (13.4% of participants). Most other AEs recorded at these later time points seemed to be unrelated to *O. volvulus* infection or IVM treatment. Three study participants experienced severe or serious AEs after the 7-day inpatient observation period. One participant experienced a severe (Grade 3) reaction to a bee sting. One participant required a limb amputation for gangrene (a SAE), and one participant had a fatal serious adverse event (liver failure because of metastatic gall bladder cancer that was diagnosed months after treatment). These severe and serious AEs were judged by study physicians and the Medical Monitor to have been unrelated to IVM treatment.

## DISCUSSION

This study has provided detailed data on IVM’s current efficacy and on the kinetics of Mf reductions in the eyes and skin in persons with light to moderate *O. volvulus* infections in a hypoendemic area in a forest-savannah transition zone in eastern Ghana. Although some might consider light infections to be a limitation of this study, our results may be more relevant to the current status of onchocerciasis in Africa than those from clinical trials that were performed in more heavily infected persons in the 1980s.^11^^,^^15^^,^^16^ That is because IVM (provided alone as CDTi or provided as MDA with albendazole in areas with coendemic lymphatic filariasis) has been very widely used in sub-Saharan Africa in recent years. For example, 152.9 million treatments were distributed in WHO’s Africa region in 2019 (70.4% of the total targeted population).^2^ Preventive chemotherapy with IVM over many years has reduced the prevalence and intensity of onchocerciasis infections in most endemic areas and interrupted transmission in some foci.^17^^,^^18^ It is interesting to compare results from this study to those from persons who were treated with IVM in a phase 3 clinical trial that compared the efficacy of IVM and moxidectin (the “moxidectin trial”).^19^ That study was conducted approximately 10 years before our study in persons with heavier parasite burdens. The geometric mean skin Mf/mg values in the moxidectin trial and in the present study were 32.2 and 12.7, respectively, and many more participants in the moxidectin trial had ≥ 10 MfAC at baseline than in the present study (15% versus 6.9%, respectively). These differences in baseline infection intensities resulted in different treatment outcomes. For example, only 11% of IVM recipients in the moxidectin trial had negative skin snips 6 months after treatment versus 54% in the present study. Indeed, 46% of the participants in our study had baseline skin Mf counts below the 10/mg required for inclusion in the moxidectin trial. We believe that this fact increases the relevance of our study for onchocerciasis during an era when WHO is aiming for elimination rather than control. We believe that relatively few areas in Africa outside of the loiasis belt currently have onchocerciasis prevalences and intensities comparable to those in the moxidectin trial study sites.

IVM was effective for clearing Mf from the skin in this study, and few participants had positive skin snips 7 days after treatment. In contrast, changes in MfAC were variable but not dramatic 7 days after treatment. Mf reappeared in skin snips in many participants between 3 and 6 months after treatment, as expected. Although a small number of participants had only slight reductions in skin Mf counts between baseline and 3 months, skin Mf counts did not increase between 3 and 6 months in those participants. Thus, suboptimal responses to IVM treatment as reported from other areas in Ghana^13^ were not common in this study.

This study yielded some unexpected results. For example, we expected MfAC to be quite uncommon in persons with skin mf counts below 20/mg. Although baseline MfAC frequencies were correlated with skin Mf counts, 27.7% of all persons in our study of persons with mostly light to moderately infections had MfAC at baseline, and MfAC was present in 15.2% of persons with baseline skin Mf counts between 3 and 5 Mf/mg. We also expected MfAC to be rare 6 months after IVM treatment in persons with light to moderate infections based on prior publications.^16^^,^^20^ Thus, we were somewhat surprised that 5.7% of participants had MfAC present 6 months after IVM treatment. Although only one participant in our study had a MfAC count > 3 in either eye 6 months after treatment, this result and the presence of MfAC 12 months after IVM in a significant number of participants in the moxidectin trial show that a single pretreatment dose of IVM does not guarantee clearance of MfAC. Indeed, several participants who were treated with IVM in the moxidectin trial had high MfAC counts after IVM that were comparable to that seen in the outlier in our study. Finally, we were also surprised by how often MfAC was detected 7 days after treatment in persons with negative baseline slit lamp exams. This suggests that a single slit lamp examination is insensitive for detecting MfAC when counts are low, as in this study. We doubt that this was caused by movement of Mf into the eyes shortly after treatment. Of course, MfAC results can vary from day to day in persons with low MfAC counts, and Mf may have been easier to see in the AC after they were paralyzed by IVM. To improve MfAC detection, our future studies will require participants to bend their head forward and further downward for at least 10 minutes just prior to slit lamp examinations.

The finding that mild or moderate AEs were common in the days following IVM treatment was consistent with prior reports.^11^^,^^15^ Detailed ophthalmological results will be reported separately. Although OCT examinations provided some interesting results, they did not detect intraocular AEs after IVM treatment.

In conclusion, this study has provided interesting data on the continued efficacy and tolerability of IVM in a population with light to moderate *O. volvulus* infections that are common in Africa at this time. Ophthalmological testing in this study included the first use of OCT in an onchocerciasis clinical trial. This IVM study provides a good foundation for studies of new treatments for onchocerciasis, and it has provided a cohort of candidates for a planned trial of a new treatment that requires pretreatment with IVM.

## Supplemental Material


Supplemental materials
