# Supplementary material for: A Reevaluation of the Tolerability and Effects of Single-Dose Ivermectin Treatment on Onchocerca volvulus Microfilariae in the Skin and Eyes in Eastern Ghana
Source: Am J Trop Med Hyg. 2021 Nov 29;106(2):740–5. doi: 10.4269/ajtmh.21-0859 (PMC8832884; doi:10.4269/ajtmh.21-0859)
Supplement: Supplementary file 1 [file tpmd210859.SD1.pdf]

Table S1. Total number of individuals reporting each AE type, during 7 days following treatment with ivermectin

AE Description	Frequency (%)
Any AE	187 (81.0%)
Itching Skin	93 (40.3%)
Headache	54 (23.4%)
Joint Or Muscle Pain	45 (19.5%)
Acute Swelling (Beyond Baseline Lymphedema)	41 (17.7%)
Abdominal Pain	24 (10.4%)
Waist Pain	19 (8.2%)
Itching, Ocular	16 (6.9%)
Rash	13 (5.6%)
Diarrhea	12 (5.2%)
Fever (Non-Axillary Temperatures Only)	11 (4.8%)
Eye Pain	10 (4.3%)
Chest Pain	9 (3.9%)
Dizziness, Giddiness, Or Fainting	9 (3.9%)
Swollen Buttocks	8 (3.5%)
Swollen Leg or Feet	7 (3%)
Chills	5 (2.2%)
Flank Pain	5 (2.2%)
Cough	4 (1.7%)
Neck Pain	4 (1.7%)
Ear Pain	3 (1.3%)
Painful Groin	3 (1.3%)
Swollen Arm Or Hand	3 (1.3%)
Toothache	3 (1.3%)
Vomiting	3 (1.3%)
Arm Pain	2 (0.9%)
Bodily Pain	2 (0.9%)
Calf Pain	2 (0.9%)
Fatigue	2 (0.9%)
Heaviness Of The Whole Body	2 (0.9%)
Mucus Stool	2 (0.9%)
Muscle Weakness	2 (0.9%)
Otitis Media	2 (0.9%)
Palpitation	2 (0.9%)
Swollen Face	2 (0.9%)
Swollen Or Painful Nodes (Armpit Or Groin)	2 (0.9%)
Throat Pain	2 (0.9%)
Watering Eyes	2 (0.9%)
Burning Sensation At Vagina Side	1 (0.4%)
General Malaise	1 (0.4%)
Hypertension	1 (0.4%)
Itching Ear	1 (0.4%)
Nausea	1 (0.4%)
Numbness of Right Limbs	1 (0.4%)
Pain Of Skin	1 (0.4%)
Swollen Scrotum	1 (0.4%)

AE Description	Frequency (%)
Upper Respiratory Tract Infection	1 (0.4%)

Percentages calculated as the number of individuals reporting the AE / total number of individuals in the study (n=231)
