# Supplementary material for: A Reevaluation of the Tolerability and Effects of Single-Dose Ivermectin Treatment on Onchocerca volvulus Microfilariae in the Skin and Eyes in Eastern Ghana
Source: Am J Trop Med Hyg. 2021 Nov 29;106(2):740–5. doi: 10.4269/ajtmh.21-0859 (PMC8832884; doi:10.4269/ajtmh.21-0859)
Supplement: Supplementary file 2 [file tpmd210859.SD2.pdf]

Supplemental Table 2. Individuals with adverse events recorded at either the 3-month or 6-month follow-up time point following treatment with ivermectin

AE Description	Frequency (%)
Any AE	127 (58.5%)
Itching, Ocular	29 (13.4%)
Abdominal Pain	18 (8.3%)
Joint Or Muscle Pain	18 (8.3%)
Headache	16 (7.4%)
Cough	8 (3.7%)
Waist Pain	8 (3.7%)
Chest Pain	7 (3.2%)
Eye Pain	6 (2.8%)
Itching Skin	6 (2.8%)
Watering Eyes	6 (2.8%)
Bodily Pain	4 (1.8%)
Diarrhea	4 (1.8%)
Malaria	4 (1.8%)
Pruritus	4 (1.8%)
Acute Swelling (Beyond Baseline Lymphedema)	3 (1.4%)
Muscle Weakness	3 (1.4%)
Otitis Externa	3 (1.4%)
Dizziness, Giddiness, Or Fainting	2 (0.9%)
Dysentery	2 (0.9%)
Fever (Non-Axillary Temperatures Only)	2 (0.9%)
Flank Pain	2 (0.9%)
Rash	2 (0.9%)
Abscess Left Big Toe	1 (0.5%)
Abscess Left Thumb	1 (0.5%)
Abscess Right 4th Finger	1 (0.5%)
Abscess Right Index Finger	1 (0.5%)
Amputation Of The 4th And 5th Toes Of The Right Foot As A Result Of Motorbike Accident	1 (0.5%)
Anorexia	1 (0.5%)
Backache	1 (0.5%)
Bee Sting	1 (0.5%)
Boil On The Left Arm	1 (0.5%)
Boil On The Left Leg	1 (0.5%)
Burning Sensation In The Legs	1 (0.5%)
Burns	1 (0.5%)
Conjunctivitis	1 (0.5%)
Difficulty Breathing (Wheezing Or Dyspnea)	1 (0.5%)
Discharge Right Ear	1 (0.5%)
Ear Pain	1 (0.5%)
Eyelid Twitching	1 (0.5%)
Facial Pain	1 (0.5%)
Gastritis	1 (0.5%)
Gastroenteritis	1 (0.5%)
Gritty Sensation	1 (0.5%)
Hypertension	1 (0.5%)
Insomnia	1 (0.5%)

AE Description	Frequency (%)
Itching Ear	1 (0.5%)
Left Testicular Pain	1 (0.5%)
Motorbike Accident With Laceration Of Left Leg	1 (0.5%)
Neck Pain	1 (0.5%)
Numbness Of Soles	1 (0.5%)
Numbness of Right Limbs	1 (0.5%)
Otitis Media	1 (0.5%)
Pain In The Hands	1 (0.5%)
Palpitation	1 (0.5%)
Pustule On The Left Arm	1 (0.5%)
Septic Laceration Right Knee And Foot	1 (0.5%)
Septic Lacerations Right Knee And Foot	1 (0.5%)
Sore On Left Leg	1 (0.5%)
Swollen Leg or Feet	1 (0.5%)
Swollen Painful Left Breast	1 (0.5%)
Swollen Right Foot As A Result Of Trauma	1 (0.5%)
Throat Pain	1 (0.5%)
Toothache	1 (0.5%)
Trauma To The Left Loin	1 (0.5%)
Upper Respiratory Tract Infection	1 (0.5%)
Viral Hepatitis	1 (0.5%)
Waist Pain/ Painful Legs	1 (0.5%)

Percentages calculated as the number of individuals reporting the AE / total number of individuals with follow-up data for AEs at 3 or 6 months (n=217)
